# Comparison of Genomes of Three *Xanthomonas oryzae *Bacteriophages

**DOI:** 10.1186/1471-2164-8-442

**Published:** 2007-11-29

**Authors:** Chia-Ni Lee, Rouh-Mei Hu, Teh-Yuan Chow, Juey-Wen Lin, Hui-Yi Chen, Yi-Hsiung Tseng, Shu-Fen Weng

**Affiliations:** 1 Institute of Molecular Biology, National Chung Hsing University, Taichung, Taiwan; 2 Department of Biotechnology, Asia University, Wufeng, Taichung, Taiwan; 3 Institute of Medical Biotechnology, Central Taiwan University of Science and Technology, Taichung, Taiwan; 4 Institute of Biochemistry, National Chung Hsing University, Taichung, Taiwan; 5 Biotechnology Center, National Chung Hsing University, Taichung, Taiwan

## Abstract

**Background:**

Xp10 and OP1 are phages of *Xanthomonas oryzae *pv. oryzae (Xoo), the causative agent of bacterial leaf blight in rice plants, which were isolated in 1967 in Taiwan and in 1954 in Japan, respectively. We recently isolated the Xoo phage Xop411.

**Results:**

The linear Xop411 genome (44,520 bp, 58 ORFs) sequenced here is 147 bp longer than that of Xp10 (60 ORFs) and 735 bp longer than that of OP1 (59 ORFs). The G+C contents of OP1 (51%) and Xop411 and Xp10 (52% each) are less than that of the host (65%). The 9-bp 3'-overhangs (5'-GGACAGTCT-3') in Xop411 and Xp10 are absent from OP1. More of the deduced Xop411 proteins share higher degrees of identity with Xp10 than with OP1 proteins, while the right end of the genomes of Xp10 and OP1, containing all predicted promoters, share stronger homology. Xop411, Xp10, and OP1 contain 8, 7, and 6 freestanding HNH endonuclease genes, respectively. These genes can be classified into five groups depending on their possession of the HNH domain (HNN or HNH type) and/or AP2 domain in intact or truncated forms. While the HNN-AP2 type endonuclease genes dispersed in the genome, the HNH type endonuclease genes, each with a unique copy, were located within the same genome context. Mass spectrometry and N-terminal sequencing showed nine Xop411 coat proteins, among which three were identified, six were assigned as coat proteins (4) and conserved phage proteins (2) in Xp10. The major coat protein, in which only the N-terminal methionine is removed, appears to exist in oligomeric forms containing 2 to 6 subunits. The three phages exhibit different patterns of domain duplication in the N-terminus of the tail fiber, which are involved in determination of the host range. Many short repeated sequences are present in and around the duplicated domains.

**Conclusion:**

Geographical separation may have confined lateral gene transfer among the Xoo phages. The HNN-AP2 type endonucleases were more likely to transfer their genes randomly in the genome and may degenerate after successful transmission. Some repeated sequences may be involved in duplication/loss of the domains in the tail fiber genes.

## Background

*Xanthomonas oryzae *pv. oryzae (Xoo) is a gram-negative plant pathogenic bacterium that causes leaf blight in rice plants, thus having a serious effect on rice production in Taiwan, China, Japan, India, and South America [1]. Agrochemicals have been somewhat effective for disease control, although biological control using bacteriophages has been considered [2]. In addition, phages that specifically infect Xoo have been used to type Xoo hosts in the field [3,4].

Among the Xoo phages are the lytic phages Xp10, Xp12, OP1, and OP2, and the filamentous phages Xf and phiXo [2-8]. Recently, the genomic sequences of Xp10 (44,373 bp, 60 ORFs), OP1 (43,785 bp, 59 ORFs), and OP2 (46,643 bp, 62 ORFs) were determined. Xp10 and OP1 both have linear genomes and share high degrees of similarity at both the nucleotide and amino acid levels [2,6]. In contrast, OP2 has a circularly permuted and terminally redundant genome, which differs in sequence from those of Xp10 and OP1 [2,6,8]. Xp15 is a phage of *X. campestris *pv. pelargonii; its genomic sequence (55,770 bp) is available in the NCBI database (AY986977).

We recently isolated a Xoo bacteriophage, Xop411, from rice plants from a rice paddy near National Chung Hsing University that showed serious symptoms of bacterial leaf blight [9]. During our sequencing of the Xop411 phage genome, the genomic sequences of Xp10 and OP1 were published [2,6]. Since comparative analysis of several bacteriophages from a single species offers a unique opportunity to study the mechanisms that drive prokaryotic genetic diversity [10], we compared the sequence of Xop411 with those of Xp10, isolated in Taiwan in 1967, and OP1, isolated in Japan in 1954 [2,4,6,7].

## Results and discussion

### Assignments of Xop411 genes

**Figure 1:**
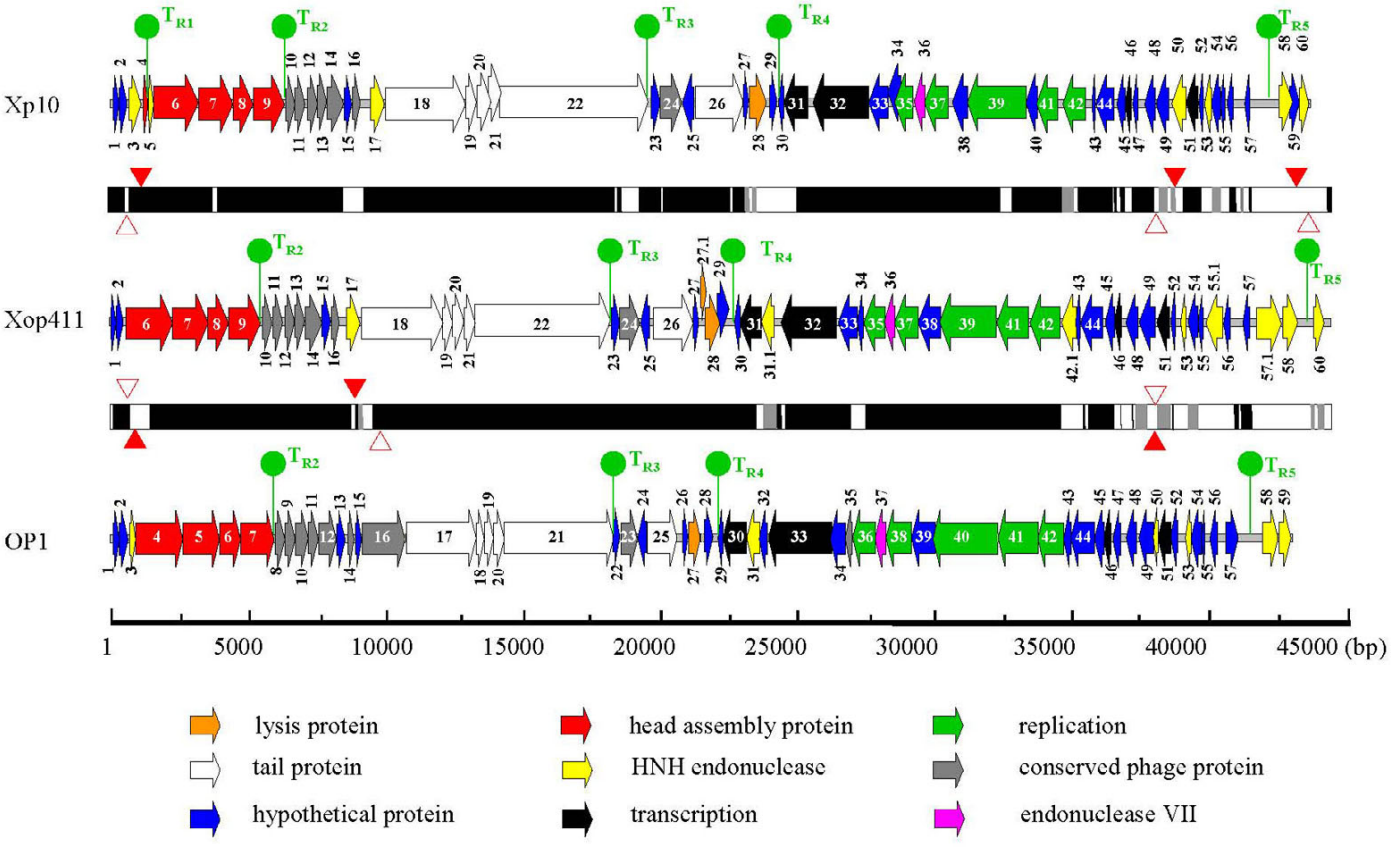
**Genomic organization of phages Xop411, Xp10 and OP1**. Colored arrows indicate the directions and categories (denoted below) of the genes. The bars between the genomic maps indicate the identities at the nucleotide level between Xop411 and Xp10 (upper) and between Xop411 and OP1 (lower); black denotes regions of > 80% identity; grey denotes regions of 65–80% identity; and white denotes regions of < 65% identity. Insertions are indicated with filled arrowheads and deletions with empty arrowheads. Knobs indicate the positions of predicted terminators.

Assembly of over 450 overlapped sequences (over 6× coverage) of the Xop411 genome showed that it was linear and consisted of 44,520 bp. The terminal sequence, 5'-GGACAGTCT-3', is identical to the 9-bp 3'-protruding sequence of Xp10 but was not observed in OP1 [2,6]. The G+C contents of the three Xoo phages were similar, 52% each for Xop411 and Xp10 and 51% for OP1 [6], but deviated from the 65% of Xoo [11]. The three phages showed highly similar genomic organization and highly similar protein products (Figure 1, Table 1). The Xp10 gene numbers were used for the corresponding genes (58) in Xop411, with a two-part number assigned when an additional gene was present, for example 31.1 for the gene between 31 and 32 (Additional file 1). Some genes, encoding HNH endonucleases (underlined) or hypothetical proteins, were only found in Xop411 (p42.1, p55.1, p57.1), Xp10 (p03, p04, p40, and p59) or OP1 (ORF15, 16, and 32), or were missing only from Xop411 (p05/ORF3, p47/ORF47, and p50/ORF50), Xp10 (p27.1/ORF26.1, p31.1/ORF31) or OP1 (p17/p17) (Figure 1, Table 1). These findings indicate that numerous insertions/deletions have occurred in the Xoo phages. More deduced Xop411 proteins shared higher degrees of similarity with Xp10 than with OP1 proteins. Only 15 Xop411 proteins, most located between p23 and p31, shared higher identities with OP1 than with Xp10 proteins (Table 1). These findings suggest that Xop411 is more closely related to Xp10 than to OP1. Although sequence information from more phages is required, discrepancies in similarity indicate that geographical separation may have limited lateral gene transfer between phages and other sources.

**Table 1:** Comparison of proteins deduced from the genes of *X. oryzae *phages Xop411, Xp10, and OP1.

Xop411		Xp10		OP1		Xop411 with Xp10	Xop411 with OP1	Xp10 with OP1
Gene	Length (aa)	Gene	Length (aa)	Gene	Length (aa)	id (%)/aligned aa	id (%)/aligned aa	id (%)/aligned aa
p01	72	p01	72	ORF1	72	98/72	88/72	88/72
p02	101	p02	101	ORF2	101	90/101	81/80	75/79
		p03	154			61/134 (p17)^*a*^		65/134 (ORF31)^*b*^
						64/130 (p31.1)^*a*^		55/134 (ORF58)^*b*^
						61/134 (p42.1)^*a*^		
						63/142 (p55.1)^*a*^		
		p04	64			100/64 (p06)^*a*^		69/56 (ORF4)^*b*^
		p05	67	ORF3	65	-	-	65/49 (ORF3)^*b*^
								51/35 (ORF58)^*b*^
p06	569	p06	561	ORF4	561	93/561	84/561	83/561
p07	432	p07	432	ORF5	432	99/432	98/432	98/432
p08	245	p08	245	ORF6	245	76/245	97/245	77/245
p09	390	p09	390	ORF7	390	98/390	97/390	98/390
p10	115	p10	115	ORF8	115	95/115	92/115	90/115
p11	124	p11	124	ORF9	125	96/124	96/123	98/123
p12	119	p12	119	ORF10	157	98/119	90/118	88/118
p13	118	p13	118	ORF11	118	96/118	91/118	94/118
p14	210	p14	210	ORF12	210	94/210	96/210	97/210
p15	100	p15	100	ORF13	100	95/100	88/100	89/100
p16	100	p16	100	ORF14	68	97/100	83/68	85/68
				ORF15	76		-	-
				ORF16	510		-	-
p17	169	p17	172			66/171 (p17)^*c*^	62/163 (ORF31)^*b*^	64/164 (ORF31)^*b*^
						65/164 (p50)^*c*^	51/168 (ORF58)^*b*^	57/169 (ORF58)^*b*^
						61/166 (p58)^*c*^		
						61/134 (p03)^*c*^		
p18	998	p18	998	ORF17	999	94/998	90/999	91/999
p19	118	p19	118	ORF18	117	98/118	96/117	94/117
p20	152	p20	152	ORF19	152	98/152	97/152	99/152
p21	130	p21	146	ORF20	146	99/130	95/130	95/146
p22	1573	p22	1574	ORF21	1571	97/1574	96/1573	96/1574
p23	96	p23	96	ORF22	100	66/96	97/96	67/96
p24	231	p24	230	ORF23	231	61/231	93/231	63/231
p25	123	p25	122	ORF24	123	97/109	96/109	95/109
p26	453	p26	498	ORF25	431	79/499	88/453	72/499
p27	69	p27	74	ORF26	69	100/62	97/69	96/62
p27.1	98	p28	223	ORF26.1^*d*^	98	93/43	96/98	96/31
p28	178	p28	223	ORF27	166	92/177	94/167	93/166
p29	111	p29	99	ORF28	111	81/77	90/111	81/79
p30	58	p30	63	ORF29	58	67/53	81/58	59/42
p31	293	p31	332	ORF30	288	71/289	77/289	80/289
p31.1	160			ORF31	167	71/157 (p50)^*c*^	68/158 (ORF31)^*b*^	69/163 (p50)^*c*^
						67/157 (p58)^*c*^	56/157 (ORF58)^*b*^	69/166 (p58)^*c*^
						65/155 (p17)^*c*^		64/164 (p17)^*c*^
						63/124 (p03)^*c*^		65/134 (p03)^*c*^
				ORF32	91		-	-
p32	746	p32	746	ORF33	774	91/746	89/746	87/746
p33	255	p33	255	ORF34	188	94/255	74/189	75/189
p34	86	p34	172	ORF35	86	98/72	97/72	96/82
p35	245	p35	245	ORF36	279	100/245	97/270	97/245
p36	134	p36	135	ORF37	135	99/116	97/116	96/116
p37	309	p37	309	ORF38	309	97/309	94/309	94/309
p38	266	p38	201	ORF39	288	98/188	93/265	91/188
p39	793	p39	794	ORF40	793	98/794	95/792	95/792
		p40	139			100/112 (p41)^*a*^		97/113 (ORF41)^*b*^
p41	374	p41	261	ORF41	438	98/260	94/372	92/260
						100/112 (p40)^*c*^		97/113 (p40)^*c*^
p42	280	p42	280	ORF42	282	82/280	85/280	85/280
p42.1	167					68/165 (p17)^*c*^	65/163 (ORF31)^*b*^	
						65/164 (p58)^*c*^	58/164 (ORF58)^*b*^	
						66/163 (p50)^*c*^		
						61/134 (p03)^*c*^		
p43	53	p43	50	ORF43	81	81/49	-	-
p44	222	p44	222	ORF44	255	89/222	78/222	81/222
p45	105	p45	104	ORF45	105	76/102	83/105	79/102
p46	73	p46	73	ORF46	73	71/73	72/73	84/73
		p47	67	ORF47	90	-	-	67/67
p48	134	p48	138	ORF48	108	71/134	53/107	70/108
p49	179	p49	151	ORF49	161	80/150	59/159	63/151
		p50	174	ORF50	52	65/164 (p17)^*a*^	-	-
						70/162 (p31.1)^*a*^		69/163 (ORF31)^*b*^
						66/163 (p42.1)^*a*^		53/163 (ORF58)^*b*^
						66/168 (p55.1)^*a*^		
p51	141	p51	144	ORF51	142	80/140	86/141	74/140
p52	50	p52	50	ORF52	72	86/50	-	-
p53	63	p53	78	ORF53	62	67/64 (p53)^*c*^	74/58 (ORF53)^*b*^	75/44 (p53)^*c*^
						43/55 (p17)^*c*^	42/42 (ORF31)^*b*^	50/36 (p58)^*c*^
						53/26 (p58)^*c*^	50/22 (ORF50)^*b*^	52/36 (p50)^*c*^
						44/43 (p50)^*c*^		45/37 (p17)^*c*^
p54	111	p54	111	ORF54	120	87/111	80/110	70/110
p55	56	p55	55	ORF55	35	67/55	62/37	88/35
p55.1	189					66/168 (p50)^*c*^	64/162 (ORF31)^*b*^	
						66/163 (p58)^*c*^	55/163 (ORF58)^*b*^	
						65/169 (p17)^*c*^		
						63/142 (p03)^*c*^		
p56	65	p56	75	ORF56	79	67/64	51/64	56/73
p57	114	p57	64	ORF57	132	-	65/102	-
p57.1	277					38/104 (p17)^*c*^	35/138 (ORF31)^*b*^	
						39/104 (p03)^*c*^	32/103 (ORF58)^*b*^	
						31/173 (p50)^*c*^		
						37/103 (p58)^*c*^		
p58	174	p58	167	ORF58	172	37/114 (p17)^*c*^	34/116 (ORF31)^*b*^	57/169 (p17)^*c*^
						35/122 (p50)^*c*^	35/110 (ORF58)^*b*^	56/167 (p58)^*c*^
						36/98 (p58)^*c*^		53/163 (p50)^*c*^
						35/98 (p03)^*c*^		55/134 (p03)^*c*^
		p59	124			-	-	-
p60	119	p60	119	ORF59	131	71/114	69/119	73/119

^*a*, *b*, ^and ^*c *^indicate sequence comparison with a protein from Xop411, OP1, and Xp10 itself, respectively. ^*d*^, predicted ORF in this study. Symbol -, no similarity was found.

Holin genes required for host lysis were not assigned for Xp10 and OP1 [2,6]. These genes are usually small and adjacent to the cognate lysozyme genes, with their protein products usually containing at least one transmembrane domain (TMD) and a hydrophilic C-terminal domain [12]. In Xop411, p27.1 (98 aa, with one TMD at aa 25–47), located upstream of the previously characterized lysozyme gene (p28) [9], was assigned as the putative holin gene. However, since p27.1 overlaps with p28 by 104 bp and lacks a hydrophilic C-terminal domain, it is unclear whether it encodes holin function. A corresponding ORF was identified in OP1, but the corresponding region in Xp10 was assigned to the N-terminus of the lysozyme gene (Table 1).

### The next best matched ORFs other than those from Xp10 and OP1

The deduced Xop411 proteins also share similarities with proteins other than those of Xp10 and OP1, and proteins encoded in five Xop411 regions are worth noting (see Additional file 2): 1) The tail-related proteins p19 to p22, encoded in a 5.9-kb region, share 33–44% identity (55–63% similarity) with ORFs of the *X. campestris *pv. pelargonii phage Xp15. 2) Proteins p26 to p28, encoded in a 2.3-kb region and including tail fiber and phage lysozyme, show 33–48% identity to proteins from *Chromobacterium violaceum*. 3) Proteins p35 to p37, encoded in a 2.1-kb region, share 30–47% identity with proteins from *Pseudomonas aeruginosa*. 4) Proteins p38 to p41, encoded in a 4.3-kb region, show 38–45% identity to proteins from *Burkholderia pseudomallei*. 5) Protein p33 shares 60% identity with a protein from *Bradyrhizobium *sp. In addition, Xop411 p08 (ClpP protease), p28 (lysozyme) and p39 (DNA polymerase I) are similar to proteins from *Xylella fastidiosa *(25–38% identity) and *X. axonopodis *pv. citri (42% identity) (see Additional file 2). These data suggest that Xoo phages have actively participated in gene transfer with several organisms. In contrast, the Xoo genome did not contain homologues with significant similarity (i.e. with expected values less than e^-4^) to the proteins of the three phages. Since the Xoo phages are lytic, opportunities to exchange genetic material with the host may have been rare.

### Gene products related to endonucleases of the HNH family

Members of the HNH endonuclease family are encoded by free-standing ORFs between genes or within introns or inteins in viruses, bacteriophages, and bacteria, as well as in eukaryotic nuclear and organellar genomes [13]. Most of these proteins are homing endonucleases involved in the mobility of their own genes or of the introns/inteins in which they are located [13-15]. These HNH proteins are characterized by the motif His-Asn-His at the N-terminus but share little overall sequence similarity and can be classified into 8 subsets [16]. Proteins of the second subset usually consist of an HNN domain and an adjacent DNA-binding domain, AP2 (Pfam:PF00847) or IENRI (Smart:SM00479), and are found primarily in phage genomes [17,18]. For example, multiple copies of HNH endonuclease genes are present in the sequenced genomes of coliphages RB16 (DQ023482-7), RB43 (NC_007023), T1 [19], Rtp [20] and T5 [21] as well as in the lactophage bIL170 [22].

**Figure 2:**
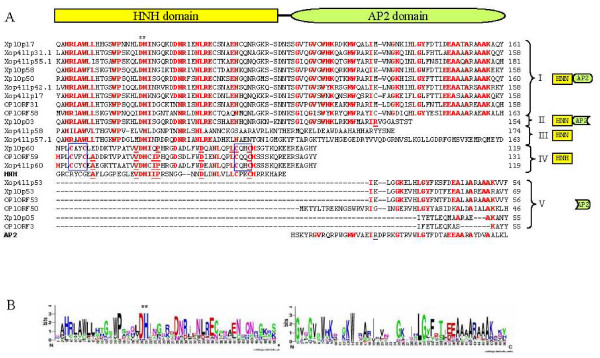
**Alignment of the 21 putative HNH endonucleases from the three Xoo phages**. (A) Sequence alignment. The conserved residues are in bold-face and the boxes indicated the cysteine dyads (CX_2_C) flanking the conserved Asp/His residue (*). (B) Consensus sequences of HNH and AP2 domains, displayed using Weblogo.

Xp10 and OP1 contain 7 (p03, p05, p17, p50, p53, p58, and p60) and 6 (ORF 3, 31, 50, 53, 58, and 59) genes encoding HNH endonucleases, respectively [2,6]. It was suggested that i) these proteins conserve many functionally important residues which may preserve their ability to bind DNA, ii) these genes may have populated the genomes through gene duplication and/or transposition, iii) their presence may account for the branched DNA structures observed by electron microscopy following denaturation and renaturation, and iv) one or more of these HNH family proteins may be involved in domain duplication of the tail fiber, which can alter the host range (see below) [2,6]. The Xop411 genome was found to contain 8 (p17, p31.1, p42.1, p53, p55.1, p57.1, p58, and p60) HNH endonuclease genes (Figures 1, 2). Using Weblogo to analyze these 21 Xoo phage proteins, consensus sequences were generated for the HNH and AP2 domains (Figure 2) [23]. They could be divided into 5 groups (Figure 2A). The HNN domain was found in proteins of groups I (9 proteins, each with intact HNN and AP2 domains), II (1 protein, with intact HNN and C-terminally truncated AP2 domains) and III (2 proteins, each containing only an HNN domain but no AP2 domain), whereas the HNH domain was detected only in the 3 proteins of group IV, which do not retain an AP2 domain. The 6 proteins in group V had degenerated, losing their HNH domains and over half of the N-terminus of their AP2 domains. A phylogenetic tree based on the alignment of 50 conserved amino acids of the HNH domain of the 15 proteins in groups I to IV suggests that the HNH type endonucleases may have arisen from an ancestor different from that of the HNN type endonucleases (see Additional file 3).

We found that all the HNN domain-containing proteins of the Xoo phages have conserved Asp/His residues flanked by two quasi-conserved boxes (HRLAWLL and WP) at the N-terminus and three conserved boxes (DNR, NLRE and EN) at the C-terminus, but do not have either metal-binding cysteine-dyads (CX_2_C) or conserved GG motifs (Figure 2A). The lack of a metal-binding motif suggests that these HNN type endonucleases may not require zinc ion to function. Since most HNN-AP2/IENRI proteins are intron-encoded site-specific endonucleases [16], the presence of multiple HNN-AP2 endonuclease genes in all three Xoo phage genomes suggests that these genes, like the homing-endonuclease genes (HEGs), are able to self-duplicate in the genome. However, since no conserved sequences could be identified in the flanking regions of these endonuclease genes and their genomic locations varied among the three phages, it is likely that transmission of these HNN-AP2 endonuclease genes was sequence-independent.

The HNH domains of the group IV proteins, which share higher degrees of similarity with the consensus HNH domain, have two cysteine-dyads (CX_2_C) flanking the conserved Asp/His residues, suggesting that zinc ion is required for their function, as well as two boxes (DX_2_NL and CH) on the C-terminal side of each domain (Figure 2A). These group IV proteins are similar to the HNH-type protein (gp13) found in the lactophage bIL170, which has two cysteine-dyads (CX_2_CX_36_CX_2_C) and no DNA-binding motif [22]. As endonucleases of this type are present as unique copies at the analogous positions of the Xoo phage genomes (the right end), they may have specific functions other than transposition, similar to the HNN-AP2 type endonucleases.

**Figure 3:**
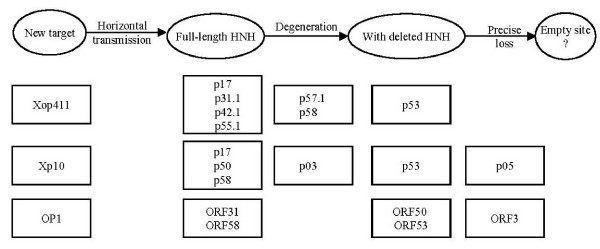
**Linear gains and losses of HNN-AP2 endonuclease genes in the three Xoo phages**. The boxes contain the possible proteins at different stages of degeneration.

The HNN-AP2 type endonuclease genes may not only be able to transmit into multiple sites of the genome but may also degenerate. For example, although the genes *hegG *and *hegJ *are present in the genomes of three T5 phage strains, our sequence analyses showed that full-length genes are retained in the strains sequenced in France (GenBank accession numbers AY692264) and Moscow (AY543070), but that both genes had degenerated in the T5 strain ATCC11303-B5 (AY587007). Specifically, a short insertion disrupted *hegG *(AAX11946 and AAX11947) and two point deletions caused frame shifts in *hegJ *(AAX12048), suggesting that degeneration of HNH endonuclease genes may occur after a deleterious insertion/deletion. In addition, one T4 HNH type Mob endonuclease gene, *mobA*, was found to have degenerated into a pseudogene [24]. A cyclical model of gain and loss of HEGs [25,26] has been used to deduce the possible evolutionary path of the I-SceI endonucleases of a self-splicing group I intron in *Saccharomyces cerevisiae *and the intron/HEG of T-even-like phages [27,28]. For Xoo phages, however, the data may be better explained by a linear model of gain and loss, in which functional alien endonuclease genes would be fixed but start degenerating after successful incorporation (Figure 3). For example, in Xp10, proteins p17, p50 and p58, each with intact HNH and AP2 domains, may represent endonuclease family members that retain their functions; proteins p03 (with a complete HNN domain but lacking the C-terminus of AP2) and p53 (retaining only the C-terminus of AP2) may represent proteins after different degrees of progressive degeneration; and protein p05, which has only a small segment of a highly degenerated AP2 domain, may represent a gene product with the greatest extent of degeneration and the most ancient HNH-AP2 endonuclease in the genome (Figure 3). Similar clues to gene degeneration were observed in the HNH-AP2 endonuclease genes of the other two phages (Figure 3).

### Promoters and terminators

We found that the nucleotide sequences between the end of p55 and the right end of the genome were highly variable in the three Xoo phages, with Xp10 and OP1 being more similar to each other than either were to Xop411, and segments with higher degrees of identity present at different positions (see Additional file 4). Mosaicism of the common segments suggests that these phages have undergone numerous recombination events, possibly during co-infections, resulting in gene rearrangements and insertion/deletion. In Xp10, the intergenic region between p57 and p58 separates the genes transcribed leftward and rightward and contains all six promoters [6,29,30]. Based on a similarity search, we located putative promoters resembling those of Xp10 in Xop411 and OP1. We found that the promoter sequences in Xp10 and OP1 were highly conserved, but shared lower degrees of identity with the Xop411 promoters (see Additional file 5). In addition, Xop411 had five sequences located between p56 and p57, and one sequence, P3, between p57.1 and p58, whereas OP1 had four sequences located between ORF57 and ORF58 and two, P_up _and φP1, contained within ORF57 (see Additional file 4).

Similarity searches of the Xop411 and OP1 genomes for the five predicted terminators of Xp10 [29] revealed four corresponding sequences at analogous positions (Figure 1, Table 2). These predicted terminators in Xop411, T_R2 _to T_R5_, each shared high degrees of identity with the respective analogous terminators in the other phages. However, sequences similar to Xp10 T_R1_, which is thought to possess a low efficiency of termination [29], were not found in Xop411 and OP1, suggesting that such a low-efficiency terminator may not be essential.

**Table 2:** Predicted terminators in Xp10, Xop411, and OP1 genome.

Terminator name	Phage	Positions	Sequence	Energy score	Tail score	Identity/aligned base
T_R1_	Xp10	1390~1411	CTGCCCTACTTATGGGCAGTTT	7.4	3.5	12/12
						
T_R2_	Xp10	6573~6603	GGGAGGGGCTGGGAAACTGGCCCCTCTCTTT	13.4	3.6	18/18
	Xop411	5704~5735	GGGAGGGGCTGGGGGAACTGGCCCCTCTCTTT	12.4	3.4	18/18
	OP1	5842~5872	GAGAGGGGCTAGGAAACTGGCCCCTCTCTTT	17.8	3.6	18/18
T_R3_	Xp10	19285~19308	GGGGCAGGGTTTCCTGCCCCATTT	15.0	3.3	16/16
	Xop411	18320~18343	GGGGCAGGGTTTCCTGCCCCATTT	15.0	3.3	16/16
	OP1	19466~19489	GGGGCAGGCTTTCCCTGCCCCTTT	14.0	4.4	16/16
T_R4_	Xp10	23718~23760	GGGAGGGAGCTAAGCCTTAATGGCCTAGCCCCTCCCTTTTTTT	15.5	5.9	28/28
	Xop411	22629~22670	ATAGGGGACCTATTGCCTTTAATGGCAGGGTCCCCTTTTTTT			24/28
	OP1	23709~23752	GGGAGGGAGCTAAGCCTTTAATGGCCTAGCCCCTCCCTTTTTT	14.5	5.7	28/28
T_R5_	Xp10	42740~42759	CTGAACGATCCGTTCAGTTT	10.9	3.5	14/14
	Xop411	43861~43880	CTGAACGGCTCGTTCAGTTT	10.9	3.6	14/14
	OP1	42375~42394	CTGAACGATCCGTTCAGTTT	10.9	3.8	14/14

### Domain duplications in tail fiber and implications in host range

Japanese isolates of Xoo can be classified into four phagovars, based on their susceptibility to OP1, with host-range mutants of OP1 capable of infecting different phagovars [2]. Sequencing of the tail fiber genes from these phage strains revealed that changes in host range are due to duplications in at least one of three domains (domains 1, 2, and 3) in ca. 118 aa at the N-terminus (see Additional file 6). This is similar to findings in other phages; for example, the host range of T4 is expanded by duplications of a small region of the tail fiber adhesin [31]. Amino acid sequence alignments showed that OP1 possesses domains -1-2-3-, Xp10 has domains -1-2-2-2-3- [2] and Xop411 exhibits domains -1-2-3-3-3- (see Additional file 6). Interestingly, while OP1 and OPh1 have the same domain architecture (-1-2-3-) and no drastic changes in the surrounding amino acid residues, OP1 infects only phagovar A whereas OP1h infects only phagovar B (see Additional file 6) [2]. This finding suggests that these related Xoo phages might use a complex structure, also containing other component(s), to determine the host range, with mutations in the latter component(s) altering the host range. Further tests are needed to understand the host ranges of Xop411 and Xp10.

In mouse minisatellite Pc-1, tandem repeats of d(GGCAG)n, which can facilitate the formation of a telomere-like intra-molecular folded-back quadruplex structure, have been shown to be hotspots of recombination during meiosis [32-34]. The genes encoding the tail fibers of the Xoo phages contain many short repeats (see Additional File 7), including i) inverted repeats that are all located outside the domains, which may be important in the acquisition/loss of domain architectures, ii) direct G-rich pentanucleotide (GGCAG) repeats at both ends of domains 1 and 2, and iii) a direct G-rich octanucleotide (CAGGCCGC) repeat flanking domain 3. It is currently unclear whether the presence of these short direct repeats can facilitate the duplication/deletion of the tail fiber domains by recombination, as observed for mouse minisatellite Pc-1. Inoue et. al. proposed that the HNH-family proteins may be involved in domain duplication via recombination using Holliday junction structures as the intermediates [2], but it is not clear if this is the mechanism occurring here.

### Identification of virion proteins

**Figure 4:**
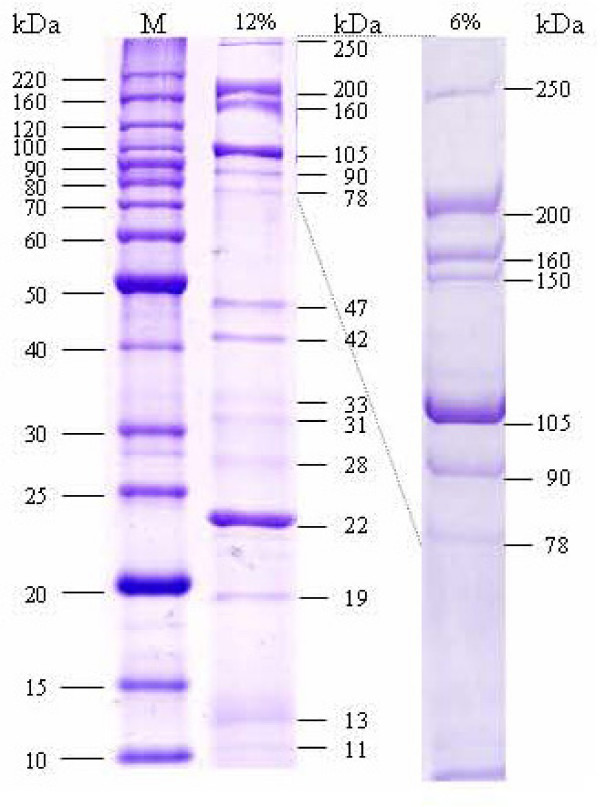
**SDS-polyacrylamide gel electrophoresis of Xop411 virion proteins**. The purified Xop411 particles were separated in 12% (middle lane) or 6% (right lane) polyacrylamide gels and stained with Coomassie brilliant blue. The proteins are named by their apparent sizes. Lane M contained molecular weight markers.

SDS-PAGE separation of the Xp10 virion proteins resulted in 6 major bands, three of which (p09, major head; p14, major tail; p26, tail fiber) were identified [6]. SDS-PAGE separation of the Xop411 virion proteins resulted in at least 16 discrete bands: 15 (of MW 250, 200, 160, 105, 90, 78, 47, 42, 33, 31, 28, 22, 19, 13, and 11 kDa) on 12% gels and 7 (of MW 250, 200, 160, 150, 105, 90, and 78 kDa) on 6% gels (Figure 4). LC MS/MS analysis (see Additional file 8) indicated that these bands contained 14 proteins, 9 from the virion and 5 from the host. The 250-, 200-, 150-, 78- and 42-kDa bands contain oligomers of p09, the 41.5-kDa major capsid subunit, of 2 to 6 subunits. Oligomerization of p09 was also observed in Xp10, but in the 140- and 165-kDa bands and in high MW materials in the gel wells. Xp10 p09 may be cleaved by a phage-encoded protease, p08, generating a mature major head protein of 283 aa, which is 170 aa less than the precursor protein [6]. In contrast, our N-terminal sequencing of the 42-kDa band gave a sequence, TDITSK, showing that only the N-terminal methionine was missing.

The head portal protein, p07, with a calculated MW of 47 kDa, was found in the 47- and 31-kDa bands, suggesting that the unprocessed and processed forms co-exist in the virions. LC-MS/MS analysis showed that the 31-kDa band contained another protein, p26, which was identified as the tail fiber in Xp10 [6]. N-terminal sequencing showed that the 22-kDa band was p14, the major tail protein in Xp10. The 13-kDa band was also a doublet, containing p10 (phage conserved protein in Xp10) and p19 (tail protein). The 160-, 105-, and 11-kDa bands were identified as p22 (tail protein), p18 (tail length tape measure protein), and p13 (phage conserved protein in Xp10), respectively. In summary, six more proteins than those identified for Xp10 were found here, and the conserved proteins p10 and p13 in Xp10 were found to be phage coat proteins.

The 5 host proteins in the 4 bands were TonB-dependent receptor FyuA (90-kDa), outer membrane protein MopB and hypothetical protein XOO0584 (33-kDa), MopB and colicin receptor protein CirA (28-kDa), and hypothetical protein XOO4199 (19-kDa). Since the experiments were repeated four times using virions freshly purified by ultracentrifugation, the consistent presence of these proteins indicates that they were rather tightly associated with the phage particles.

## Conclusion

Our results, showing that Xop411 and Xp10 have the same G+C content and that more of the deduced Xop411 proteins share higher degrees of identity with Xp10 than with OP1 proteins, indicate that the two phages isolated in Taiwan are more closely related to each other than they are to OP1. Thus, geographical separation may have limited lateral gene transfers between phages and other sources. However, our finding that more of the DNA sequences are conserved by Xp10 and OP1 in the region between p55 and the right end of the genome, a region containing the predicted promoters, suggests that Xop411 has undergone sequence rearrangements and insertions/deletions to a greater degree. The HNN-AP2 type endonucleases may have transferred their genes randomly and begun degenerating after successful horizontal transmission, whereas the HNH type endonucleases, each with one copy, were located within the same genome context. Comparison of the host range and the architecture of the duplicated domains in the N-terminus of the tail fiber proteins suggests that the Xoo phages may need additional components for adsorption. Some of the repeated sequences in and around the domains may be involved in duplication/loss of the domains. We identified 6 more proteins than those identified for Xp10, with p10 and p13 shown to be phage coat proteins.

## Methods

### Bacteria, bacteriophages, and growth conditions

*X. oryzae *pv. oryzae (*Xoo*) was cultivated in Tryptic Soy Broth or Agar (Bacto™) at 28°C and *Escherichia coli *was grown in LB medium at 37°C. Ampicillin (50 μg/ml) was added when necessary. The procedures described previously [9] were used for plaque assay, phage propagation (using Xoo strain 21 as the host), purification of phage particles, and isolation and restriction enzyme digestion of phage DNA.

### Sequence analyses

The purified phage DNA was treated in a HydroShear (GeneMachines, San Carlos, CA). Fragments of 1.0 to 3.0 kb were isolated and ligated into the *Eco*RV site of pBluescript II SK. Clones were randomly picked and subjected to nucleotide sequencing (ABI 3700). To determine the 3'-protruding terminal sequences (gap closure), the Xop411 genomic DNA was treated with or without Klenow enzyme, using its 3'→5' exonuclease activity and ligated using T4 ligase, and the ligation products were PCR-amplified separately with a pair of primers annealed close to the ends, followed by sequencing of the amplicons. Thus the extra nucleotides, obtained from the PCR product amplified on the template that had not been treated with Klenow enzyme, represented the 3'-protruding sequence. A+T content was analyzed by using the program available online [35]. DNA sequences were assembled using the SeqMan program from the DNASTAR package (DNASTAR, Madison, WI) and analyzed with NCBI software [36]. ORF was predicted using GeneMark. The nucleotide sequence of phage Xop411 has been deposited in GenBank under accession no. DQ777876.

HNH endonucleases were identified by searching for conserved domains as well as similarities to the endonucleases identified in Xp10 [6]. The BLAST program was used to search for nucleotide and amino acid similarities, and phylogenetic analysis was performed using the parsimony method (Phylip package ver. 3.66). Bootstrap values were obtained for a consensus based on 1000 randomly generated trees using SEQBOOT and CONSENSE.

### Sodium dodecyl sulfate-polyacrylamide gel electrophoresis (SDS-PAGE) and LC-MS/MS analysis

Phage particles purified by ultracentrifugation were mixed with sample buffer, heated in a boiling water bath for 3 min, and subjected to SDS-PAGE separation in 12% or 6% (w/v) polyacrylamide gel. Protein bands were visualized by staining the gels with Coomassie brilliant blue, excised from the gels and subjected to LC-MS/MS (ABI Qstar System) analysis at the Biotechnology Center, National Chung Hsing University.

### N-terminal amino acid sequencing of proteins

The proteins from the Xop411 particles separated in SDS-PAGE were transferred to polyvinylidene difluoride membranes and stained with Coomassie brilliant blue. Membrane strips containing the isolated protein bands were excised and subjected to Edman degradation to determine their N-terminal sequences (477A sequencer, PE Applied Biosystems).

## Authors' contributions

CNL, RMH, YHT, and SFW participated in genome analysis, data interpretation and manuscript preparation. CNL and HYC performed the SDS-PAGE and LC MS/MS analysis. RMH, TYC and JWL participated in the study design and data interpretation and helped to draft the manuscript. All the authors have read and approved the final manuscript.

## Supplementary Material

Additional file 1 Gene assignment of the Xoo phage Xop411. Click here for file

Additional file 2 Similarities shared between the Xop411 proteins and those of *Xylella *and *Xanthomonas*. Click here for file

Additional file 3 Phylogenetic tree based on alignments of 50 conserved amino acids from the HNH domains of proteins in groups I to IV. Click here for file

Additional file 4 **The region between p55 and the right end of genome of the three Xoo phages**. Thick arrows indicate the direction and length of the genes. Corresponding genes are in the same colors, except that yellow indicates an additional gene. The horizontal bars represent percent identity of the nucleotide sequence, with black denoting > 80%, grey 65–80%, and white < 65% identity. Blocks A, B, C, D, and E of Xop411 and Xp10 (total, 842 bp) showed 71–92% identity; blocks F, G, H, and I of Xop411 and OP1 (total, 669 bp) showed 77–88% identity; and blocks J, K, L, M, N, O, and P of Xp10 and OP1 (total, 1,636 bp) showed 81–94% identity. The horizontal red lines indicate AT-rich regions of Xp10 (262 bp, nt 42,929–43,190 with 68% A+T), Xop411 (240 bp, nt 43,921-44,160 with 72% A+T, including an 80-bp internal segment of 96% A+T from nt 43,974-44,033 with 4 perfect 15-bp tandem direct repeats ATTATTAATATTTAT), and OP1 (336 bp, nt 42,631-42,966 with 63% A+T). These AT-rich regions are worth testing for the possibility of containing replication origins of the Xoo phages. Bent arrows and knobs represent the predicted promoters and terminators, respectively. Click here for file

Additional file 5 **Alignment of promoters found in Xp10 and predicted for OP1 and Xop411**. Bases identical to those of the Xp10 are on black background. Ratios to the right are bases matched to the Xp10 sequences. Color of the bases: blue, -35; green, conserved to Xp10 RNA polymerase promoters; orange, extended -10 promoter elements; pink, -10; yellow, transcription start sites. The extended -10 elements were found to be resistant to p7, the inhibitor of transcription initiation, in Xp10. Click here for file

Additional file 6 **Domain duplication at the N-terminus of the deduced tail fiber proteins of Xoo phages**. (A) Alignment of the sequence of Xop411 p26 with its homologues in Xp10, OP1, and four OP1 host range mutants. A domain is indicated by a line above the sequences with an Arabic number in circle. Different amino acid residues within the duplicated domains are shaded. (B) Summary of domain duplications in the tail fiber proteins of Xop411, Xp10, OP1 and host range mutants of OP1. The relationships between domain duplication (number inside circle) and phagovars (letter in parenthesis) infected by OP1 phage strains are shown. Scheme representations are after Inoue et al [6]. Click here for file

Additional file 7 **Positions of the 2 direct and 4 inverted repeats in the duplicated domains of the tail fiber genes from Xop411 (A), Xp10 (B), OP1 (C), OP1hc (D), OP1h (E), OP1h2 (F), and OP1h2c (G)**. Shown are DNA regions containing the duplicated domains and the flanking sequences. Domains are in different colors: 1, red; 2, blue; 3, green. When the same domain runs consecutively, the alternate one(s) (i.e., 2^nd ^and 4^th ^if any) is underlined. Direction and position of the repeats are indicated by half arrows above the sequences. Direct repeats (DR) are mostly located inside the domains especially in the domain junctions, whereas all inverted repeats (IR) are outside the domains. Click here for file

Additional file 8 Identification of Xop411 virion proteins by mass spectrometry. Click here for file
