# Supplementary material for: Comparison of Genomes of Three Xanthomonas oryzae Bacteriophages
Source: BMC Genomics. 2007 Nov 29;8:442. doi: 10.1186/1471-2164-8-442 (PMC2248197; doi:10.1186/1471-2164-8-442)
Supplement: Additional file 1 — Gene assignment of the Xoo phage Xop411. [file 1471-2164-8-442-S1.pdf]

Additional file1: Gene assignment of *Xamthomonas oryzae* phage Xop411.

Xop411 Gene [a]	Start	Stop	G+C/ 3rd (%)[b]	Length (aa)	Function	The next best match ORF other than that from Xp10 and OP1 (identity%, gi number)
+p01	111	329	56/49	72	hypothetical protein	
+p02	326	631	61/65	101	hypothetical protein	
+p06	743	2452	54/62	569	terminase large subunit	Z6045 in *E. coli* O157:H7 EDL933 prophage CP-933P (48%, 15801961)
+p07	2462	3760	53/54	432	head portal protein	DaciDRAFT_2373 in *Delftia acidovorans* SPH-1 phage HK97 family (47%, 118730339)
+p08	3769	4506	51/53	245	protease of the ClpP family	S34 in *Delftia acidovorans* SPH-1 phage HK97 family (43%, 118730338)
+p09	4525	5697	59/63	390	head protein	TM1040_1668 in *Silicibacter* sp. TM1040 phage HK97 family (47%, 99081509)
+p10	5745	6092	50/50	115	phage coat protein	gp7 in *Burkholderia* phage phi1026b (34%, 38707897)
+p11	6092	6466	50/47	124	head-tail joining protein	Aave_1775 in *Acidovorax avenae* subsp. citrulli AAC00-1 (38%, 120610456)
+p12*	6570	6929	52/51	119	conserved phage protein	BAV1467 in *Bordetella avium* 197N (36%, 115422554)
+p13	6926	7282	51/50	118	phage coat protein	BAV1468 in *Ralstonia solanacearum* GMI1000 (31%, 115422555)
+p14	7305	7937	57/62	210	major tail	BP3369 in *Bordetella pertussis* Tohama I (33%, 33594257)
+p15	7940	8242	52/62	100	hypothetical protein	
+p16*	8251	8553	53/58	100	conserved phage protein	DaciDRAFT_0025 in *Delftia acidovorans* SPH-1 (32%, 118734511)
+p17*	8858	9367	50/57	169	HNH endonuclease	ORF257 in *Salmonella* phage Felix 01 (42%, 38707764)
+p18	9408	12404	54/55	998	tail length tape measure protein	Sputw3181_2470 in *Shewanella* sp. W3-18-1 lambda family phage (42%, 38707764)
+p19	12401	12757	47/50	118	tail protein	T in *Xanthomonas campestris* pv. pelargonii phage Xp15 (41%, 66392069)
+p20	12754	13212	46/49	152	tail protein	U in *Xanthomonas campestris* pv. pelargonii phage Xp15 (33%, 66392125)
+p21	13214	13606	47/45	130	peptidoglycan hydrolase	V in *Xanthomonas campestris* pv. pelargonii phage Xp15 (36%, 66392070)
+p22	13591	18312	51/49	1573	tail protein	W in *Xanthomonas campestris* pv. pelargonii phage Xp15 (44%, 66392071)
+p23	18357	18647	45/38	96	hypothetical protein	
+p24	18647	19342	47/38	231	conserved phage protein	RSc1699 in *Ralstonia solanacearum* GMI1000 (26%, 17546418)
-p25	19710	19339	53/63	123	hypothetical protein	
+p26	19828	21189	52/51	453	tail fiber	CV0418 in *Chromobacterium violaceum* ATCC 12472 (33%, 34495873)
+p27	21192	21401	46/49	69	hypothetical protein	
+p27.1	21419	21715	51/52	98	putative holin	
+p28	21612	22148	54/59	178	phage-type lysozyme	CV0727 in *Chromobacterium violaceum* ATCC 12472 (48%, 34496182)<br>SSON_2437 in *Shigella sonnei* Ss046 (45%, 74312894)
+p29	22117	22452	52/46	111	hypothetical protein	
-p30	22846	22670	49/60	58	hypothetical protein	

-p31	23717	22836	53/60	293	DNA ligase	
-p31.1*	24196	23714	53/63	160	HNH endonuclease	YE2335 in *Yersinia enterocolitica* subsp. enterocolitica 8081 (41%, 123442580)
-p32	26774	24534	53/57	746	T7-like RNA polymerase	AGR_C_2186 in *Agrobacterium tumefaciens* str. C58 (34%, 15888521)
-p33	27568	26801	57/59	255	hypothetical protein	BradDRAFT_0915 in *Bradyrhizobium* sp. BTAi1 (60%, 78699704)
-p34*	27825	27565	44/44	86	hypothetical protein	Rsph17029_0652 in *Rhodobacter sphaeroides* ATCC 17029 (43%, 126461423)
-p35	28572	27835	54/69	245	DNA polymerase I	BURPS1710b_1651 in *Pseudomonas aeruginosa* strain LAK1 (47%, 76810908)
-p36	29065	28661	54/57	134	packaging and recombination endonuclease VII	gp33 in *Pseudomonas aeruginosa* strain LAK1 (39%, 114796463)
-p37	29997	29068	53/54	309	DNA polymerase I	gp22 in *Pseudomonas aeruginosa* strain LAK16 (30%, 114796401)
-p38	30807	30010	52/58	266	hypothetical protein	BURPS1710b_1650 in *Burkholderia pseudomallei* 1710b (45%, 76809154)
-p39	33191	30810	51/58	793	DNA polymerase I	BURPS1710b_1649 in *Burkholderia pseudomallei* 1710b (44%, 76810071)
-p41	34302	33181	54/64	374	replicative helicase	BURPS1710b_1647 in *Burkholderia pseudomallei* 1710b (38%, 76811336)
-p42	35336	34494	55/60	280	DNA primase	ORF14 in *Pseudomonas aeruginosa* LKD16 (28%, 116077876)
-p42.1	36069	35566	50/57	167	HNH endonuclease	ECs3504 in *Escherichia coli* O157:H7 (41%, 15832758)
-p43	36205	36044	54/48	53	hypothetical protein	
-p44	36890	36222	55/51	222	hypothetical protein	Bcep1808_1157 in *Burkholderia vietnamiensis G4* (34%, 134295268)
-p45	37313	36996	54/55	105	hypothetical protein	
-p46	37531	37310	53/66	73	inhibitor of transcription initiation and anti-terminator	
-p48	38179	37775	46/41	134	hypothetical protein	
-p49	38743	38204	50/56	179	hypothetical protein	
-p51*	39242	38817	50/56	141	peptidase of the HslV family	orf27 in *Salmonella enterica phage KS7* (32%, 62327334)
-p52	39428	39276	49/67	50	hypothetical protein	
-p53	39798	39607	54/70	63	HNH endonuclease	
-p54	40190	39855	55/58	111	hypothetical protein	
-p55	40363	40193	48/51	56	hypothetical protein	
-p55.1	41023	40454	53/60	189	HNH endonuclease	ORF257 in *Salmonella* phage Felix 01 (32%, 62327334
-p56	41257	41060	50/57	65	hypothetical protein	
+p57	41802	42146	45/46	114	hypothetical protein	
+p57.1*	42165	42998	48/54	277	HNH endonuclease	
+p58	43029	43553	51/57	174	HNH endonuclease	BDAG_01666 in *Burkholderia dolosa* AUO158 (29%, 124895048)
+p60*	44090	44449	56/63	119	HNH endonuclease	Mpe_A0538 in *Methylibium petroleiphilum* PM1 (51%, 124265731)

[a] Asterisk indicates the gene to be using GTG as the start codon. "+" denotes the positive strand, whereas "-" the complementary strand.
[b] Percentage of G + C content for the gene and the third position in codons.
