# Supplementary material for: Comparison of Genomes of Three Xanthomonas oryzae Bacteriophages
Source: BMC Genomics. 2007 Nov 29;8:442. doi: 10.1186/1471-2164-8-442 (PMC2248197; doi:10.1186/1471-2164-8-442)
Supplement: Additional file 2 — Similarities shared between the Xop411 proteins and those of Xylella and Xanthomonas. [file 1471-2164-8-442-S2.pdf]

Additional file 2: Similarities shared between the Xop411 proteins and those of *Xylella* and *Xanthomonas*.

Xop411 protein	Strain	Accession number	Functional annotation	Identity with Xop411 (%)	*E*-value
p08	*Xylella fastidiosa* Ann-1	ZP_00680226	peptidase S14	29	9e-16
	Xylella fastidiosa Dixon	ZP_00652933	peptidase S14	29	1e-15
	Xylella fastidiosa 9a5c	NP_297801	hypothetical protein XF0511	31	4e-15
p28	*Xylella fastidiosa* 9a5c	NP_297803	phage-related endolysin	34	6e-13
	Xylella fastidiosa Ann-1	ZP_00681324	phage-related endolysin	34	2e-13
	Xylella fastidiosa Dixon	ZP_00652935	glycoside hydrolase	34	2e-13
	Xylella fastidiosa Temecula-1	NP_779208	phage-related lysozyme	38	6e-13
	Xanthomonas axonopodis pv. citri str. 306	NP_641407	phage-related lysozyme	42	7e-16
p39	*Xylella fastidiosa* Temecula-1	NP_778627	DNA polymerase I	25	1e-13
	Xylella fastidiosa Ann-1	ZP_00681639	DNA-directed DNA polymerase	25	1e-13
	Xylella fastidiosa 9a5c	NP_298393	DNA polymerase I	25	2e-13
	Xylella fastidiosa Dixon	ZP_00652340	DNA polymerase A	25	1e-13
