# Supplementary figures and images for: Comparison of Genomes of Three Xanthomonas oryzae Bacteriophages
Source: BMC Genomics. 2007 Nov 29;8:442. doi: 10.1186/1471-2164-8-442 (PMC2248197; doi:10.1186/1471-2164-8-442)

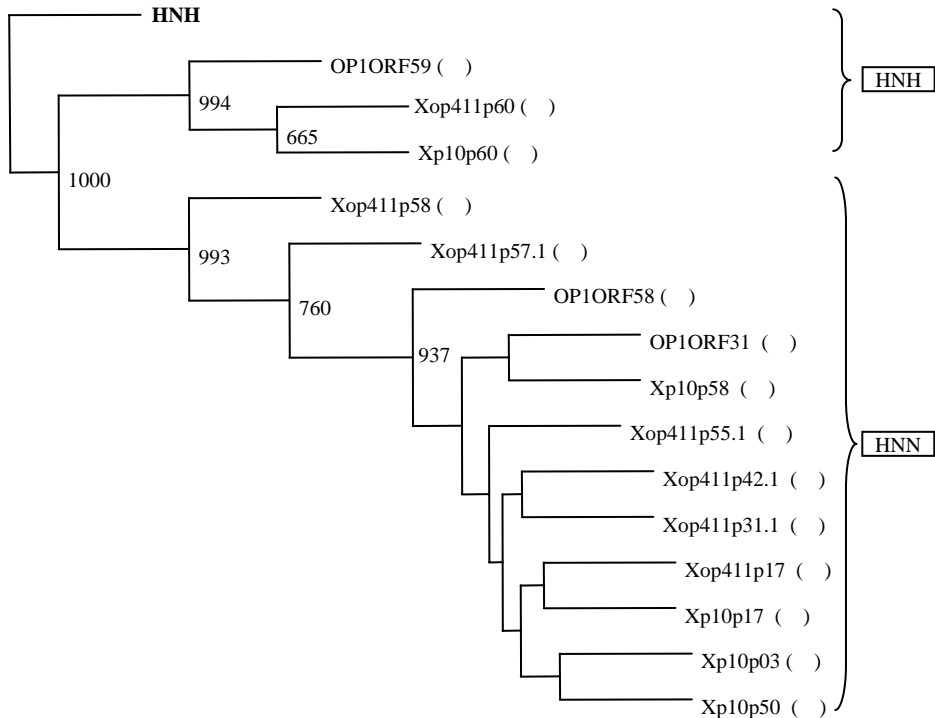

Supplement: Additional file 3 — Phylogenetic tree based on alignments of 50 conserved amino acids from the HNH domains of proteins in groups I to IV. [file 1471-2164-8-442-S3.pdf]

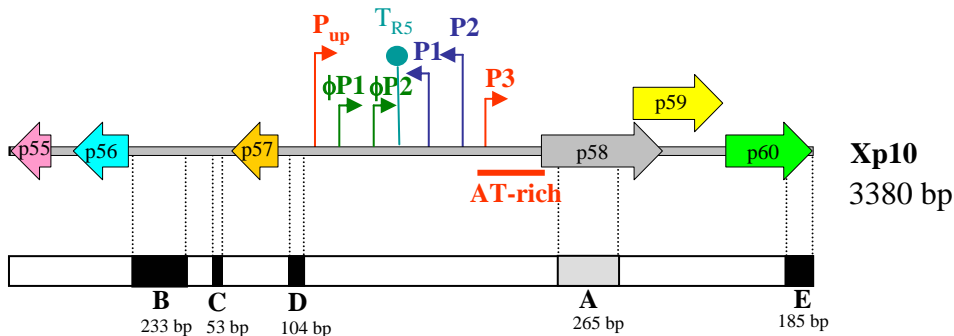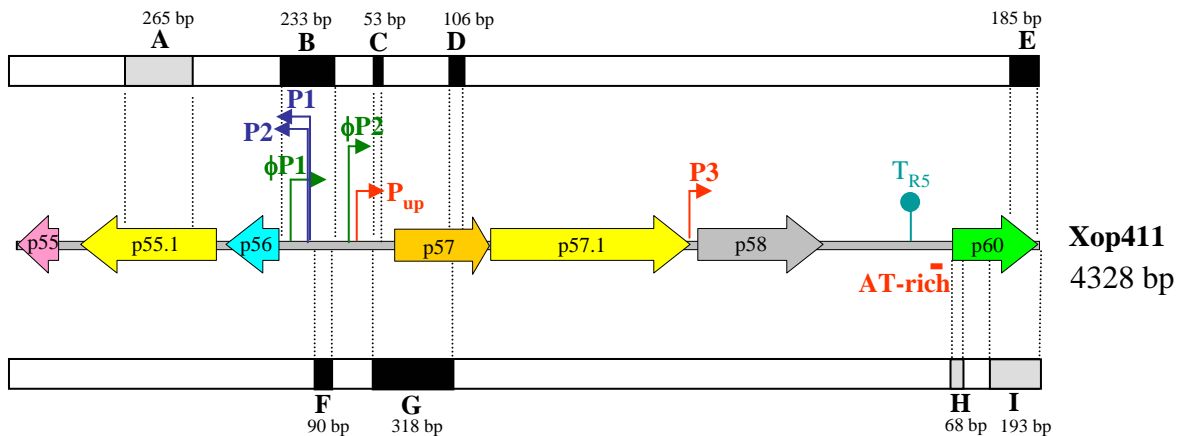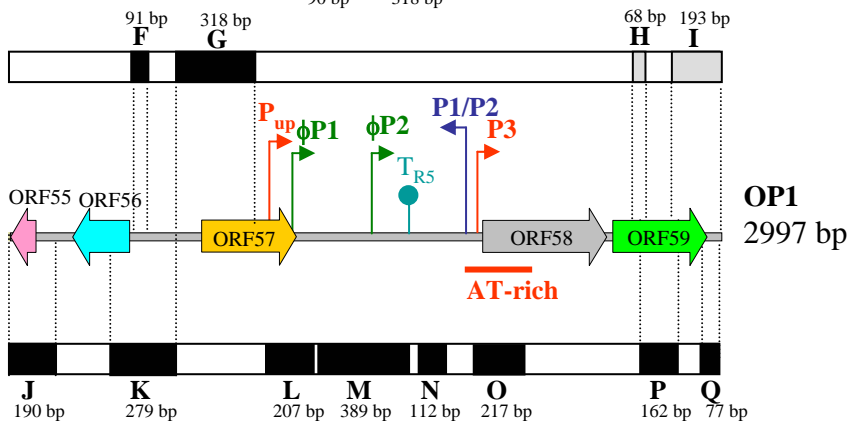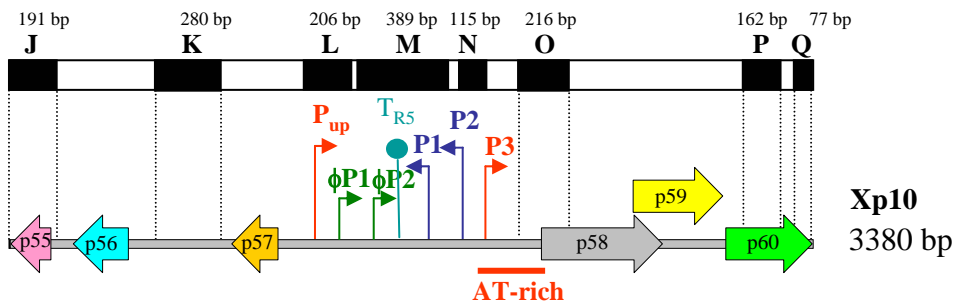

Supplement: Additional file 4 — The region between p55 and the right end of genome of the three Xoo phages. Thick arrows indicate the direction and length of the genes. Corresponding genes are in the same colors, except that yellow indicates an additional gene. The horizontal bars represent percent identity of the nucleotide sequence, with black denoting > 80%, grey 65–80%, and white < 65% identity. Blocks A, B, C, D, and E of Xop411 and Xp10 (total, 842 bp) showed 71–92% identity; blocks F, G, H, and I of Xop411 and OP1 (total, 669 bp) showed 77–88% identity; and blocks J, K, L, M, N, O, and P of Xp10 and OP1 (total, 1,636 bp) showed 81–94% identity. The horizontal red lines indicate AT-rich regions of Xp10 (262 bp, nt 42,929–43,190 with 68% A+T), Xop411 (240 bp, nt 43,921-44,160 with 72% A+T, including an 80-bp internal segment of 96% A+T from nt 43,974-44,033 with 4 perfect 15-bp tandem direct repeats ATTATTAATATTTAT), and OP1 (336 bp, nt 42,631-42,966 with 63% A+T). These AT-rich regions are worth testing for the possibility of containing replication origins of the Xoo phages. Bent arrows and knobs represent the predicted promoters and terminators, respectively. [file 1471-2164-8-442-S4.pdf]
