# Supplementary material for: Comparison of Genomes of Three Xanthomonas oryzae Bacteriophages
Source: BMC Genomics. 2007 Nov 29;8:442. doi: 10.1186/1471-2164-8-442 (PMC2248197; doi:10.1186/1471-2164-8-442)
Supplement: Additional file 5 — Alignment of promoters found in Xp10 and predicted for OP1 and Xop411. Bases identical to those of the Xp10 are on black background. Ratios to the right are bases matched to the Xp10 sequences. Color of the bases: blue, -35; green, conserved to Xp10 RNA polymerase promoters; orange, extended -10 promoter elements; pink, -10; yellow, transcription start sites. The extended -10 elements were found to be resistant to p7, the inhibitor of transcription initiation, in Xp10. [file 1471-2164-8-442-S5.pdf]

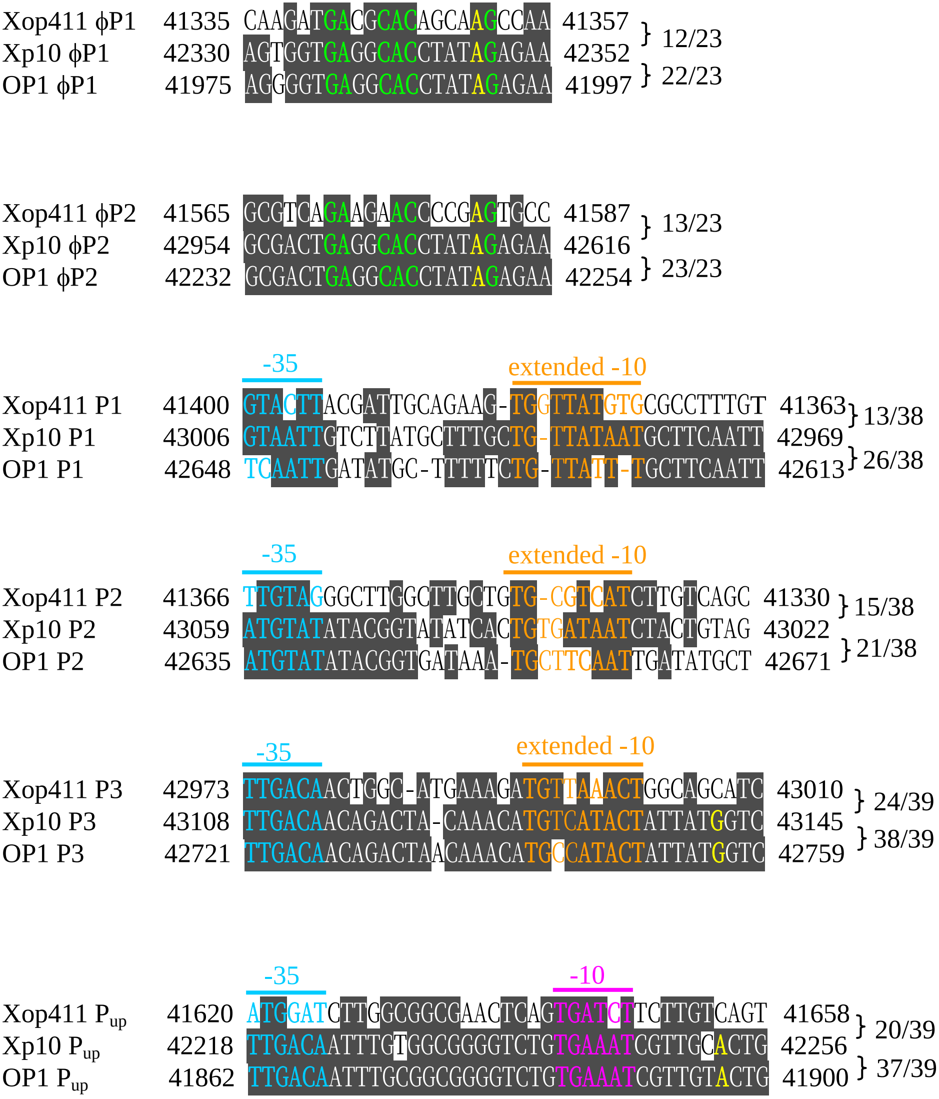

Xop411 ϕP1 41335 CAAGATGACGCACAGCAAGCCAA 41357
Xp10 ϕP1 42330 AGTGGTGAGGCACCTATAGAGAA 42352
OP1 ϕP1 41975 AGGGGTGAGGCACCTATAGAGAA 41997
} 12/23
} 22/23
Xop411 ϕP2 41565 GCGTCAGAAGAACCCCGAGTGCC 41587
Xp10 ϕP2 42954 GCGACTGAGGCACCTATAGAGAA 42616
OP1 ϕP2 42232 GCGACTGAGGCACCTATAGAGAA 42254
} 13/23
} 23/23
-35
extended -10
Xop411 P1 41400 GTACTTACGATTGCAGAAG-TGGTTATGTGCGCCTTTGT 41363
Xp10 P1 43006 GTAATTGTCTTATGCTTTGCTG-TTATAATGCTTCAATT 42969
OP1 P1 42648 TCAATTGATATGC-TTTTCTG-TTATT-TGCTTCAATT 42613
} 13/38
} 26/38
-35
extended -10
Xop411 P2 41366 TTGTAGGGCTTGGCTTGCTGTG-CGTCATCTTGTCAGC 41330
Xp10 P2 43059 ATGTATATACGGTATATCACTGTGATAATCTACTGTAG 43022
OP1 P2 42635 ATGTATATACGGTGATAAA-TGCTTCAATTGATATGCT 42671
} 15/38
} 21/38
-35
extended -10
Xop411 P3 42973 TTGACAACTGGC-ATGAAAGATGTTAAACTGGCAGCATC 43010
Xp10 P3 43108 TTGACAACAGACTA-CAAACATGTCATACTATTATGGTC 43145
OP1 P3 42721 TTGACAACAGACTAACAAACATGCCATACTATTATGGTC 42759
} 24/39
} 38/39
-35
-10
Xop411 P_{up} 41620 ATGGATCTTGGCGGCGAACTCAGTGATCTTCTTGTCAGT 41658
Xp10 P_{up} 42218 TTGACAATTTGTGGCGGGGTCTGTGAAATCGTTGCACTG 42256
OP1 P_{up} 41862 TTGACAATTTGCGGCGGGGTCTGTGAAATCGTTGTACTG 41900
} 20/39
} 37/39
