# Supplementary material for: Comparison of Genomes of Three Xanthomonas oryzae Bacteriophages
Source: BMC Genomics. 2007 Nov 29;8:442. doi: 10.1186/1471-2164-8-442 (PMC2248197; doi:10.1186/1471-2164-8-442)
Supplement: Additional file 6 — Domain duplication at the N-terminus of the deduced tail fiber proteins of Xoo phages. (A) Alignment of the sequence of Xop411 p26 with its homologues in Xp10, OP1, and four OP1 host range mutants. A domain is indicated by a line above the sequences with an Arabic number in circle. Different amino acid residues within the duplicated domains are shaded. (B) Summary of domain duplications in the tail fiber proteins of Xop411, Xp10, OP1 and host range mutants of OP1. The relationships between domain duplication (number inside circle) and phagovars (letter in parenthesis) infected by OP1 phage strains are shown. Scheme representations are after Inoue et al [6]. [file 1471-2164-8-442-S6.pdf]

A

```
                                                                  ①                        ①
Xop411   MAIVNIDLDTPRPDGKPGDDARVAFGKVNGNFADVQSQITAEVSARQSAISAVQTTVAAKASSSDLAAEISAR---------------------- 73
Xp10     MAIVNIDLDTPRPDGKPGDDARVAFSKVNANFADVQSQITAEVSARQSAISSVQTTVAAKATSSDLAAEISAR---------------------- 73
OP1      MAIVNIDLDTPRPDGKPGDDARAAFSKVNSNFADVQSQITAEVSARQSAISSVQTTVATKASSSDLASEISAR----------------------73
OP1hc    MAIVNIDLDTPRPDGKPGDDARAAFSKVNSNFADVQSQITAEVSARQSAISSVQTTVATKASSSDLASEISARQSAISSVQTTVATKASSSDLAS-95
OP1h     MAIVNIDLDTPRPDGKPGDDARVAFGKVNGNFADVQSQITAEVSARQSAISAVQTTVAAKASSSDLAAEISAR---------------------- 73
OP1h2    MAIVNIDLDTPRPDGKPGDDARVAFSKVNANFADVQSQITAEVSARQSAISSVQTTVASKATSSDLAAEISAR---------------------- 73
OP1h2c   MAIVNIDLDTPRPDGKPGDDARVAFSKVNANFADVQSQITAEVSARQSAISSVQTTVASKATSSDLAAEISAR---------------------- 73

((((                  ①                          ①                          ②
Xop411   ------------------------------------------------------------QSADNAISATVTAQGQAINQRALSTDLTAEATAR---107
Xp10     ------------------------------------------------------------QSADNAISSTVTAQGQAINQRALSTDLSAEIAAR---107
OP1      ------------------------------------------------------------QSADSAISATVTAQGQAINQRALSADLTAEVAAR---107
OP1hc    EISARQSAISSVQTTVATKASSSDLASEISARQSAISSVQTTVATKASSSDLASEISARQSADSAISATVTAQGQAINQRALSADLTAEVAAR---188
OP1h     ------------------------------------------------------------QAADNAISATVTAQGQAINQRALSTDLTAEATAR---107
OP1h2    ------------------------------------------------------------QSADNAISATVTAQGQAINQRALSTDLSAEIAAR---107
OP1h2c   ------------------------------------------------------------QSADNAISATVTAQGQAINQRALSTDLSAEIAAR---107

                    ①                          ②                          ②
Xop411   ------------------------------------------------------------------------------------------ 107
Xp10     ---------------------------QSADNAISSTVTAQGQAINQRALSTDLSAEIAARQSADNAISSTVTAQGQAINQRALSTDLSAEIAAR 175
OP1      ------------------------------------------------------------------------------------- --- 107
OP1hc    ------------------------------------------------------------------------------------- --- 188
OP1h     ------------------------------------------------------------------------------------- --- 107
OP1h2    ------------------------------------------------------------------------------------- --- 107
OP1h2c   QSAISSVQTTVASKATSSDLAAEISARQSADNAISATVTAQGQAINQRALSTDLSAEIAAR------------------------- --- 168

              ③          ③          ③
Xop411   QAADASIQATLQAADASIQATLQAADASIQATLQAADTALGARIDDVLSMVGRNRLLNPGFTRASRGPGGTFSNFTTEFYTADQWVISGVSMSGS 202
Xp10     QAADAALQAAQ----------------------QTADTALGARIDDVLSMTGRNRLLNPGFSRASRGPGGTFSNFTTEFYAVDQWVLSGVSMSGS 248
OP1      QAADVALQTTL----------------------QAADTALGSRIDGVLSMTGRNKLLNPGFTRTSRGPGGTFSNFTTEFYTVDQWVLSGISMSGS 180
OP1hc    QAADVALQTTL----------------------QAADTALGSRIDGVLSMTGRNKLLNPGFTRTSRGPGGTFSNFTTEFYTVDQWVLSGISMSGS 261
OP1h     QAADASIQATL----------------------QAADTALGARIDDVLSMVGRNRLLNPGFTRASRGPGGTFTNFTTEFYAVDQWVLSGVSMSGS 180
OP1h2    QAADASIQATLQAADASIQATL-----------QAADTALGARIDDVLSMVGRNRLLNPGFTRASRGPGGTFTNFTTEFYAVDQWVLSGVSMSGS 191
OP1h2c   QAADASIQATLQAADASIQATL-----------QAADTALGARIDDVLSMVGRNRLLNPGFTRASRGPGGTFTNFTTEFYAVDQWVLSGASMSGS 252

Xop411   WGRNQLNGASNTLAAGRNYFAVNITSANLASVGQKIEGVHTLAGSKATLSVWLRSTVAGKRLGVRIYQSFGTGGSPSAAVSTIITPTPLTLSTTW 297
Xp10     WGRNQLNGASNTLAAGRNYFAVNITAGSLASVGQKIEGVHTLAGGKATLSVWLRSTVAGKRLGVRIYQSFGTGGSPSAAVSTIITPTPLTLSATF 343
OP1      WGRNQLNGASNTLAEGRNYFVVNITSANLASVGQKIEGVHTLAGSKATLSVWLRSTVAGKRLGFRIYQSFGTGGSPSAAVSTIITPTPLTLSTTW 275
OP1hc    WGRNQLNGASNTLAEGRNYFVVNITSANLASVGQKIEGVHTLAGSKATLSVWLRSTVAGKRLGFRIYQSFGTGGSPSAAVSTIITPTPLTLSTTW 356
OP1h     WGRNQLNGASNTLAAGRNYFAVNITAGSLASVGQKIEGVHTLAGGKATLSVWLRSTVAGKRLGVRIYQSFGTGGSPSAAVSTIITPTPLTLSTNF 275
OP1h2    WGRNQLNGASNTLAAGRNYFAVNITAGSLASVGQKIEGVHTLAGGKATLSVWLRSTVAGKRLGVRIYQSFGTGGSPSAAVSTIITPTPLTLSTNF 286
OP1h2c   WGRNQLNGASNTLAAGRNYFAVNITAGSLASVGQKIEGVHTLAGGKATLSVWLRSTVAGKRLGVRIYQSFGTGGSPSAAVSTIITPTPLTLSTNF 347

Xop411   QKYTLTFDVPSVSGKTIGTNNNDHLYVVFDITNTDSYGGALSGQTGQIELAYPQLEKGSVATEFEFRAPGVEKALCEWYLKAGVVALRGNGTGSA 392
Xp10     QKYTLTFDVPSVSGKTLGTNNNDYLYLVFDIAG-DAYGTALSGQTAQIEFAFPQLEKGSVATDFELRSPGVEKALCEWYLKAGVVALRGNGTGRA 437
OP1      QKYTLTFDVPSVSGKTIGTNNNDHLYVVFDITNTDSYGGSLSGQTGQIEFAFPQLEKGSVATEFEFRAPGVEKALCEWYLKAGVVALRGNGTGSA 370
OP1hc    QKYTLTFDVPSVSGKTIGTNNNDHLYVVFDITNTDSYGGSLSGQTGQIEFAFPQLEKGSVATEFEFRAPGVEKALCEWYLKAGVVALRGNGTGRA 451
OP1h     QKYTLTFDVPSVSGKTLGTNNNDYLYLVFDITG-DAYGTALSGQTAQIEFAFPQLEKGSVATDFEFRSPGVEKALCEWYLKAGVVALRGNGTGSA 369
OP1h2    QKYTLTFDVPSVSGKTLGTNNNDYLYLVFDITG-DAYGTALSGQTAQIEFAFPQLEKGSVATDFEFRSPGVEKALCEWYLKAGVVALRGNGTGSA 380
OP1h2c   QKYTLTFDVPSVSGKTLGTNNNDYLYLVFDITG-DAYGTALSGQTAQIEFAFPQLEKGSVATDFEFRSPGVEKALCEWYLKAGVVALRGNGTRSA 441

Xop411   GAGTFISHEMRAAPAITFANTIYYYNCSGINYASYPTGVEVLVNATGTFAFYSTYIMSAEL 458
Xp10     GAGTFISHEMRAAPAITFANTIYYYNCSGINYASHTTGVEVLVNATGTFAFYSTYIMSAEL 498
OP1      GAGTFISHEMRAAPAITFANTIYYYNCSGINYASYPTGVEVLVNATGTFAFYSTYIMSAEL 431
OP1hc    GAGTFISHEMRAAPAITFANTIYYYNCSGINYASYPTGVEVLVNATGTFAFYSTYIMSAEL 512
OP1h     GAGTFIAHEMRASPAVTFANTVYYYNCSGINYVAYPTGVEVLVNATGTFAFYSTYIMSAEL 430
OP1h2    GAGTFIAHEMRASPAVTFANTIYYYNCSGINYAPYPTGVEVLVNATGTFAFYSTYIMSAEL 441
OP1h2c   GAGTFIAHEMRASPAVTFANTIYYYNCSGINYAPYPTGVEVLVNATGTFAFYSTYIMSAKL 502
```

B

Phage	Duplication	Phagovar
Xop411	--①--②--③--③--③--	
Xp10	--①--②--②--②--③--	
OP1	--①--②--③--	(A)
OP_{1hC}	--①--①--①--①--②--③--	(A, C)
OP_{1h}	--①--②--③--	(B)
OP_{1h2}	--①--②--③--③--	(A, B, D)
OP_{1h2C}	--①--②--①--②--③--③--	(A, B, C, D)
