# Supplementary material for: Comparison of Genomes of Three Xanthomonas oryzae Bacteriophages
Source: BMC Genomics. 2007 Nov 29;8:442. doi: 10.1186/1471-2164-8-442 (PMC2248197; doi:10.1186/1471-2164-8-442)
Supplement: Additional file 7 — Positions of the 2 direct and 4 inverted repeats in the duplicated domains of the tail fiber genes from Xop411 (A), Xp10 (B), OP1 (C), OP1hc (D), OP1h (E), OP1h2 (F), and OP1h2c (G). Shown are DNA regions containing the duplicated domains and the flanking sequences. Domains are in different colors: 1, red; 2, blue; 3, green. When the same domain runs consecutively, the alternate one(s) (i.e., 2nd and 4th if any) is underlined. Direction and position of the repeats are indicated by half arrows above the sequences. Direct repeats (DR) are mostly located inside the domains especially in the domain junctions, whereas all inverted repeats (IR) are outside the domains. [file 1471-2164-8-442-S7.pdf]

(A) Xop411 (domain 1-2-3-3-3)

IR$_1$
ATGGCAATTGTTAACATCGACTTAGATACACCTCGTCCAGATGGAAAACCTGGGGACgacaacg
cccgTGTGGCATTTGGAAAGGTGAACGGGAACTTCGCGGACGTCCAATCCCAGATCACTGCC
IR$_2$ DR$_1$
GAagtgtctgcTAggcagTCGGCGATATCTGCTGTGCAGACCACCGTGGCAGCAAAGGCATCA
DR$_1$ DR$_1$
TCCTCTGATCTAGCggcagAGATTTCGGCACggcagAGTGCGGACAACGCTATCTCCGCTA
CGGTCACAGCTCAAGGACAGGCCATCAACCAGCGTGCGCTTAGCACAGATTTGACggc
DR$_1$ DR$_2$
agAGGCAACAGCAAggcaggccgcAGACGCGTCAATACAGGCGACACTACAGGCCGCAGAC
DR$_2$ DR$_2$
GCGTCAATACAGGCGACACTAcaggccgcAGACGCGTCAATACAGGCGACACTAcaggccgcag
IR$_2$
acactGCTCTAGGCGCACGAATTGACGATGTGCTGAGCATGGTGGGTCGGAACAGGCTGCTCA
IR$_1$
ACCCAGGATTTACTcgggcgtc

(B) Xp10 (domain 1-2-2-2-3)

ATGGCAATTGTGAACATCGACTTAGATACACCTCGTCCGGATGGAAAACCTGGGGACGAC
IR$_3$ DR$_1$
GCCCGTGTGGCAttcagcaaGGTTAATGCTAACTTTGCTGACGTCCAATCCCAGATCACggcagA
DR$_1$
AGTTTCGGCAAggcagTCGGCAATATCTTCTGTGCAGACCACAGTGGCAGCAAAGGCTA
DR$_1$
CATCCTCTGACCTTGCAGCAGAGATTTCGGCACggcagAGTGCGGACAACGCTATCTCC
TCCACGGTCACAGCACAAGGACAGGCCATCAACCAGCGTGCCCTTAGCACTGACCTA
DR$_1$
TCAGCTGAGATCGCCGCAAggcagAGTGCGGACAACGCTATCTCCTCCACGGTCACAG
CACAAGGACAGGCCATCAACCAGCGTGCCCTTAGCACTGACCTATCAGCTGAGATCG
DR$_1$
CCGCAAggcagAGTGCGGACAACGCTATCTCCTCCACGGTCACAGCACAAGGACAGGC
DR$_1$ DR$_2$
CATCAACCAGCGTGCCCTTAGCACTGACCTATCAGCTGAGATCGCCGCAAggcaggccgc
AGACGCGGCACTACAGGCGGCGCAGCAGACAGCAGACACCGCTCTAGGCGCACGTATT
IR$_3$
GATGATGTGTTGTCAATGACGGGCCGTAACAGGttgctgaa

(C) OP1 (domain 1-2-3)

ATGGCAATTGTGAACATCGACTTAGATACACCTCGTCCAGATGGAAAACCTGGGGACGACG
IR$_3$
CTCGTGCGGCAttcagcaaGGTTAATTCAAACTTCGCGGACGTCCAATCCCAGATCACTGCCGA
DR$_1$
AGTGTCTGCCAggcagTCAGCAATATCTTCTGTGCAGACCACAGTGGCAACAAAGGCTTC
DR$_1$
CTCCTCTGACCTTGCCTCAGAGATTTCGGCCAggcagAGTGCCGACAGTGCCATCTCCG
DR$_1$
CCACGGTCACAGCACAAGggcagGCTATCAACCAGCGCGCACTCAGTGCCGATTTGACG
DR$_1$ DR$_2$ DR$_2$
GCTGAGGTCGCCGCAAggcaggccgcGGACGTGGCACTACAGACCACACTAcaggccgcAGAT
IR$_3$
ACTGCTTTGGGATCCCGCATTGATGGCGTTTTGTCCATGACTGGTCGTAACAAAttgctgaa

(D) OP1hc (domain 1-1-1-1-2-3)

ATGGCAATTGTGAACATCGACTTAGATACACCTCGTCCAGATGGAAAACCTGGGGACGACG
IR$_3$
CTCGTGCGGCAttcagcaaGGTTAATTCAAACTTCGCGGACGTCCAATCCCAGATCACTGCCGA
DR$_1$
AGTGTCTGCCAggcagTCAGCAATATCTTCTGTGCAGACCACAGTGGCAACAAAGGCTTC
DR$_1$
CTCCTCTGACCTTGCCTCAGAGATTTCGGCCAggcagTCAGCAATATCTTCTGTGCAGAC
DR$_1$
CACAGTGGCAACAAAGGCTTCCTCCTCTGACCTTGCCTCAGAGATTTCGGCCAggcagT
CAGCAATATCTTCTGTGCAGACCACAGTGGCAACAAAGGCTTCCTCCTCTGACCTTGC
DR$_1$
CTCAGAGATTTCGGCCAggcagTCAGCAATATCTTCTGTGCAGACCACAGTGGCAACAA
DR$_1$
AGGCTTCCTCCTCTGACCTTGCCTCAGAGATTTCGGCCAggcagAGTGCCGACAGTGCC

ATCTCCGCCACGGTCACAGCACAAGggcagGCTATCAACCAGCGCGCACTCAGTGCCGA
TTTGACGGCTGAGGTCGCCGCAAggcaggcccgcGGACGTGGCACTACAGACCACACTAcag
gccgcAGATACTGCTTTGGGATCCCGCATTGATGGCGTTTTGTCCATGACTGGTCGTAACAAAtt
gctgaa

(E) OP1h (domain 1-2-3)

ATGGCAATTGTGAACATCGACTTAGATACACCTCGTCCAGATGGAAAACCTGGGGACgacgcc
cgTGTGGCATTTGGAAAGGTGAACGGGAACTTCGCGGACGTCCAATCCCAGATCACTGCCG
AAGTGTCTGCTAggcagTCGGCGATATCTGCTGTGCAGACCACCGTggcagCAAAGGCATCA
TCCTCTGATCTAGCggcagAGATTTCGGCCAggcagGCTGCCGACAATGCCATTTCCGCCA
CGGTCACAGCACAAGggcagGCTATCAACCAGCGTGCGCTTAGCACAGATTTGACggcag
AGGCAACAGCAAggcaggccgcAGACGCGTCAATACAGGCGACACTAcaggccgcTGACACTGC
CCTAGGCGCACGCATTGACGATGTGCTTAGCATGGTTGGCCGTAACAGGTTGCTGAACCCA
GGATTTACTcgggcgtc

(F) OP1h2 (domain 1-2-3-3)

ATGGCAATTGTGAACATCGACTTAGATACACCTCGTCCGGATGGAAAACCTGGGGACgacgc
ccgTGTGGCAttcagcaaGGTTAATGCGAACTTtgctgacgTCCAATCCCAGATCACggcagAAGTGTC
GGCAAggcagTCGGCAATATCTTCTGTGCAGACCACAGTGGCATCAAAGGCTACATCCT
CTGACCTTGCAGCAGAAATTTCGGCACggcagAGTGCGGACAACGCTATCTCCGCCAC
GGTCACAGCACAAGGACAGGCCATCAACCAGCGTGCCCTTAGCACCGACCTATCAGC
TGAGATCGCCGCAAggcaggccgcAGACGCGTCAATACAGGCGACACTACAGGCCGCAGA
CGCGTCAATACAGGCGACACTAcaggccgcTGACACTGCCCTAGGCGCACGCATTGACGAT
GTGCTTAGCATGGTTGGCCGTAACAGGttgctgaaCCCAGGATTTACTcgggcgtcTCGTGGCCCAG
GGGGTACCTTTACGAATTTCACCACAGAGTTTTATGCTGTCGACCAGTGGGTGCTTAGTGG
cgtcagca

(G) OP1h2c (domain 1-2-1-2-3-3)

ATGGCAATTGTGAACATCGACTTAGATACACCTCGTCCGGATGGAAAACCTGGGGACgacgcc
cgTGTGGCAttcagcaaGGTTAATGCGAACTTTGCTGACGTCCAATCCCAGATCACggcagAAGTGT
CGGCAAggcagTCGGCAATATCTTCTGTGCAGACCACAGTGGCATCAAAGGCTACATCCT
CTGACCTTGCAGCAGAAATTTCGGCACggcagAGTGCGGACAACGCTATCTCCGCCACG
GTCACAGCACAAGGACAGGCCATCAACCAGCGTGCCCTTAGCACCGACCTATCAGCT
GAGATCGCCGCAAggcagTCGGCAATATCTTCTGTGCAGACCACAGTGGCATCAAAGGC
TACATCCTCTGACCTTGCAGCAGAAATTTCGGCACggcagAGTGCGGACAACGCTATCT
CCGCCACGGTCACAGCACAAGGACAGGCCATCAACCAGCGTGCCCTTAGCACCGACC
TATCAGCTGAGATCGCCGCAAggcaggccgcAGACGCGTCAATACAGGCGACACTAcaggccgc
AGACGCGTCAATACAGGCGACACTAcaggccgcTGACACTGCCCTAGGCGCACGCATTGACG
ATGTGCTTAGCATGGTTGGCCGTAACAGGttgctgaaCCCAGGATTTACTcgggcgtc
