# Supplementary material for: Comparison of Genomes of Three Xanthomonas oryzae Bacteriophages
Source: BMC Genomics. 2007 Nov 29;8:442. doi: 10.1186/1471-2164-8-442 (PMC2248197; doi:10.1186/1471-2164-8-442)
Supplement: Additional file 8 — Identification of Xop411 virion proteins by mass spectrometry. [file 1471-2164-8-442-S8.pdf]

Additional file 8 : Identification of the Xop411 virion proteins by mass spectrometry.

Size in gel (kDa)	Xop411	Xp10	Protein identification	Peptide sequence matched or coverage (%)
	Protein / kDa	Protein/ kDa		
250	p09 / 42	p09 / 41	major head protein	QILSDAPQLASYMNNR<br>GTGANDGLLGLIPQATTYAAPTTIAGATR<br>DANNQYLIGNAR<br>LALVVYRPEALISGSFA (18%)
200	p09 / 42	p09 / 41	major head protein	QILSDAPQLASYMNNR<br>DANNQYLIGNAR (7%)
160	p22 / 167	p22 / 167	tail protein	LSDQTLTIVR<br>ASASALQTLSTK (1%)
150	p09 / 42	p09 / 41	major head protein	QILSDAPQLASYMNNR<br>GTGANDGLLGLIPQATTYAAPTTIAGATR<br>DANNQYLIGNAR<br>LALVVYRPEALISGSFA (18%)
105	p18 / 106	p18 / 105	tail length tape measure protein	QSVQSLVTEVDK<br>ALEDQTQDLK<br>NYYAGLR<br>GTEVEVSSIQK<br>SGAIAVQIQSLQER (5%)
90[a]	–	–	TonB-dependent receptor FyuA	GGSASTFTSNAPGGIINFISK<br>YDVNAPYVALTFDSGK<br>LLFGVVR<br>TQEQNFEITSQR<br>SYEAHGVELEASYR<br>QADVVWQLTPSYR (10%)
78	p09 / 42	p09 / 41	major head protein	LALVVYRPEALISGSFA (4%)
47	p07 / 47	p07 / 47	head portal protein	33%
42	p09 / 42	p09 / 41	major head protein	QILSDAPQLASYMNNR<br>DANNQYLIGNAR<br>LALVVYRPEALISGSFA (11%)
33[a]	–	–	outer membrane MopB protein	LTNDAPFVTLGLGK<br>AGVGLQTTFDK (6%)
			hypothetical protein XOO0584	FALPDSADADGITADGR (10%)
31	p07 / 47	p07 / 47	head portal protein	FLTDDQYDSFAK<br>SLGLNPVDAQLLQSR (6%)
	p26 / 48	p26 / 52	tail fiber	NQLNGASNTLAAGR<br>ATLSVWLR<br>YTLTFDVPSVSGK (7%)
28[a]	–	–	outer membrane MopB protein	LTNDAPFVTLGLGK<br>GWNPYLLFGLGYQR<br>AGVGLQTTFDK<br>GVNFDFNK<br>ATTVYDYLTK (15%)
	–	–	colicin I receptor CirA	TTDLDTVNVIAK<br>LLDGALPAQFGDR<br>TAAVVDITTK<br>NGLENPVDSLNPLHDK<br>VGLNYLFGSGLR<br>DGGGIGVFAPQWAPR (11%)
22	p14 / 22	p14 / 22	major tail	ASTSSVNLLTTR<br>LEYSAGTLR (1%)
19[a]	–	–	hypothetical protein XOO4199	GVDLGDNFYGR<br>LNQNWGINGDIR<br>MDGDGNKEWSVGPR (18%)
13	p10 / 13	p10 / 13	phage coat protein	HLQAELYEDDER<br>DYVMQQLLPAAR (20%)
	p19 / 14	p19 / 14	tail protein	IGAITVLKPGLVIR (11%)
11	p13 / 14	p13 / 14	phage coat protein	67%

[a] Identified according to the sequenced genome of *Xanthomonas oryzae* pv. oryzae KACC10331
